# Control of vaccine preventable diseases in Australian infants: reviewing a decade of experience with DTPa-HBV-IPV/Hib vaccine

**DOI:** 10.1080/21645515.2020.1764826

**Published:** 2020-06-23

**Authors:** Julianne Bayliss, Michael Nissen, Damita Prakash, Peter Richmond, Kyu-Bin Oh, Terry Nolan

**Affiliations:** a Medical Affairs, GSK, Melbourne, Australia; b Scientific Affairs & Public Health, GSK, Singapore, Singapore; c Division of Paediatrics and Centre for Child Health Research, University of Western Australia, Wesfarmers Centre of Vaccines and Infectious Diseases, Telethon Kids Institute, Perth Children’s Hospital, Perth, Australia; d Medical Affairs, GSK, Singapore, Singapore; e Vaccine and Immunisation Research Group (Virgo), University of Melbourne, School of Population and Global Health and Murdoch Children’s Research Institute, Melbourne, Australia

**Keywords:** Australia, combination vaccines, coverage, infant vaccination, safety, DTPa-HBV-IPV/Hib, hepatitis B, *Haemophilus influenzae* type b, pertussis

## Abstract

The combined vaccine against diphtheria, tetanus, pertussis, hepatitis B, poliomyelitis, and *Haemophilus influenzae* b (DTPa-HBV-IPV/Hib, Infanrix Hexa, GSK) has been used for childhood immunization in Australia according to a two-, four-, six-month schedule since 2009. We reviewed data available in the Australian National Notifiable Diseases Surveillance System, annual vaccination coverage reports, the Database of Adverse Event Notifications, and peer-reviewed literature to assess vaccine coverage rates, incidence of all six vaccine preventable diseases, and the safety profile of DTPa-HBV-IPV/Hib vaccine in Australian infants over a period of ten years of exclusive use. Between 2009 and 2018 vaccine coverage for infants aged 12 months increased from 91.7% to 94.0% and from 84.9% to 92.6% for all and for Indigenous infants, respectively. Over the same time period, there were no reports of poliomyelitis, diphtheria or tetanus in infants <12 months of age. The incidence of hepatitis B among Australian infants <12 months of age remains 10 to 20-fold lower than the national average. Control of *Haemophilus influenzae* b (Hib) and pertussis disease has continued to be challenging. Timely administration of the primary series, as well as increasing coverage rates, particularly among Indigenous children, has contributed to improvements in Hib and pertussis disease control. The incorporation of additional strategies such as adjustment of the first vaccination encounter to six weeks of age, parental cocooning, and most recently maternal vaccination has further reduced the burden of pertussis, particularly during the first six months of life. The frequency of the ten most common adverse events related to the DTPa-HBV-IPV/Hib vaccine demonstrates an acceptable safety profile. Data collected over ten years of consistent, exclusive use of the DTPa-HBV-IPV/Hib vaccine in Australia highlights combination vaccination as a cornerstone in maintaining infant health.

## Introduction

### Combination vaccines for pediatric vaccination

Combination vaccines, consisting of a single injection containing two or more antigens against multiple diseases, represent a valuable approach to improving vaccine coverage, timeliness of administration, and compliance.^1^^,2^ Combination vaccines may also assist with alleviating concerns about administering multiple injections during a single healthcare visit.^2–5^ Reducing the number of injections has additional economic benefits by decreasing administration and stocking costs as well as the number of healthcare visits needed for completing the vaccination course.^6^

The combined diphtheria, tetanus, pertussis, hepatitis B, poliomyelitis, and *Haemophilus influenzae* type b vaccine (DTPa-HBV-IPV/Hib, Infanrix Hexa, GSK) was first licensed in Europe in 2000.^5,7^ DTPa-HBV-IPV/Hib is indicated for primary vaccination of infants according to a two- or three-dose schedule, followed by a booster dose given at least six months after the last dose of the primary course.

### Introduction of DTPa-HBV-IPV/Hib vaccination in Australia

The DTPa-HBV-IPV/Hib vaccine was progressively introduced onto the Australian National Immunisation Programme (NIP) as a three-dose primary vaccination series, given at two, four, and six months of age from 2005 (Table 1). The Australian Capital Territory, New South Wales, Tasmania, and Western Australia were the first to include the vaccine on their immunization programs in November 2005, followed by Victoria, Queensland, and South Australia in March 2008. Since October 2009, all Australian children have been eligible to receive the DTPa-HBV-IPV/Hib primary vaccination course on the NIP, including medically stable infants born prematurely, i.e. before 37 weeks of gestation, or infants with a low birth weight, i.e. lower than 2,500 g.^8^

In Australia, national recommendations for vaccination are developed by the Australian Technical Advisory Group on Immunisation (ATAGI), a ministerially appointed advisory group comprised of individuals with a mixture of research, clinical and implementation expertise.^9^ Vaccination recommendations are published in the Australian Immunisation Handbook (AIH)^8,9^ and includes a list of funded and recommended (private) vaccines for all populations. Funded vaccines together constitute the Australian NIP which aims to establish a good immunity base, providing infants with protection against vaccine preventable diseases through childhood, into adolescence and adulthood.^10^ The Federal Department of Health has set an aspirational target of 95% coverage for Australian infants.^11,12^ Uptake is driven in part through a nationalized register providing vaccination status on individuals and population coverage which is accessible to all registered healthcare professionals and through the development of legislation which prevents collection of government family tax benefits and enrollment of children into childcare without proof of vaccination.^13–15^

Vaccine safety is continuously monitored by the Therapeutic Goods Administration (TGA) throughout the life cycle of implementation. The TGA collates all adverse events reported for any therapeutic product in a publicly available national database. To date, more than 9.6 million doses of DTPa-HBV-IPV/Hib vaccine have been supplied to Australian children, allowing for substantial post-marketing safety analysis (Internal communication, 2019).

**Table 1.** Comparison of the Australian vaccination schedules in 1997 and 2019 for children up to one year of age

	1997		2019	
Age	Diseases	Number of vaccines administered	Diseases	Number of vaccines administered
Birth			Hepatitis B	1
2 months	Diphtheria Tetanus Pertussis Poliomyelitis Hib	3	Diphtheria Tetanus Pertussis Hepatitis B Poliomyelitis Hib Pneumococcal Rotavirus	3
4 months	Diphtheria Tetanus Pertussis Poliomyelitis Hib	3	Diphtheria Tetanus Pertussis Hepatitis B Poliomyelitis Hib Pneumococcal Rotavirus	3
6 months	Diphtheria Tetanus Pertussis Poliomyelitis Hib*	3 or 2*	Diphtheria Tetanus Pertussis Hepatitis B Poliomyelitis Hib	1
12 months	Measles Mumps Rubella Hib*	1* or 2	Measles Mumps Rubella Pneumococcal Meningococcal**	3

Hib: *Haemophilus influenzae* type b

* Hib oligosaccharide conjugate vaccine was given at 2, 4 and 12 months of age or Hib outer-membrane protein complex vaccine was given at 2, 4, 6 and 18 months of age.

**Serogroups A, C, W & Y.

Here we review ten years of use of the DTPa-HBV-IPV/Hib vaccine in Australian children, presenting surveillance data for all six diseases, with the aim of demonstrating continued improvement in vaccine coverage and sustained control of disease within the infant population. We further highlight the substantial gains achieved in Indigenous children as well as the importance of continued improvements in this at-risk population. A summary of ten years of safety data captured through the TGA reporting framework is also presented. Finally, we discuss three strategies which have been implemented in Australia as a means of reducing the burden of pertussis disease in infants during the first months of life; (i) shifting of administration of the first pertussis-containing vaccine dose from two months to six weeks of age, (ii) vaccination of household members and caregivers of new-borns (cocooning); and (iii) implementation of maternal vaccination.

## Methods

Data were gathered through a series of non-systematic reviews of available literature and summary data assessing four areas of DTPa-HBV-IPV/Hib vaccine implementation and safety in Australia (Table 2):

1. Vaccination coverage for Indigenous and non-Indigenous children, measured at 12 months of age
2. Notifications of six vaccine preventable conditions as covered by the DTPa-HBV-IPV/Hib vaccine from the national registry
3. Non-systematic review of published literature detailing the impact of DTPa-HBV-IPV/Hib vaccination in Australian infants
4. Collation of adverse event reporting data from the TGA database for adverse event reporting

**Table 2.** Source of data for disease notifications, vaccine coverage and adverse events following vaccination with DTPa-HBV-IPV/Hib vaccine in Australia

Type of data	Period	Population	Source
Disease notifications/Incidence rates	2009–2018	Overall/<1 yoa	Data from NNDSS^16^
Pertussis notifications/Incidence rates	2006–2018	By month of age for infants <1 yoa	Data from NNDSS^16^
Vaccine coverage	2009–2018	Overall/Indigenous	AIR^12^
Adverse events	2009–2018	Overall	DAEN^17^

AIR: Australian Immunisation Register; DAEN: Database of Adverse Event Notifications; NNDSS: National Notifiable Diseases Surveillance System; yoa: years of age

### Data sources

#### Vaccination coverage

Historical (2009 to 2018) childhood vaccination coverage rates for one-year-old infants, i.e. the percentage of children of 12 to less than 15 months of age in Australia who had received all of the recommended vaccines for their age, were retrieved from the Australian Immunisation Register (AIR) through the Department of Health website.^12^ We conducted non-systematic review of peer-reviewed scientific literature using the search terms “immunisation”, “Australia”, “Indigenous”, “coverage”, to collect additional data about DTPa-HBV-IPV/Hib vaccine coverage rates and timeliness of dose administration.

#### Notifications of vaccine preventable disease

The Australian National Notifiable Diseases Surveillance System (NNDSS) was established in 1990. NNDSS coordinates the surveillance of more than 50 communicable diseases or disease groups at national level. Notifications are collected by State/Territory health authorities for transmission to the Australian Government. The Australian Department of Health collates and anonymizes data, providing a daily update available through the NNDSS website.^16^ Notifications by month of age in infants less than one year old for the six vaccine-preventable diseases included in the DTPa-HBV-IPV/Hib vaccine were extracted from the Australian NNDSS data, obtained upon request to the Department of Health (Table 2).^16^ We obtained population estimates, total and infants aged less than one year stratified by month of age, from the Australian Bureau of Statistics.^18^

#### DTPa-HBV-IPV/Hib vaccine impact

We conducted a non-systematic review of peer-reviewed literature using the search terms “immunisation”, “Australia”, “Indigenous”, “infant”, “hepatitis B”, “*Haemophilus influenzae*” and “pertussis”, to assess the impact of the DTPa-HBV-IPV/Hib vaccine on the Australian population between 2009 and 2018.

Pertussis notifications in infants <12 months of age were further considered according to the prevailing recommendation for providing infant protection against disease. Between 2006 and 2009, infant vaccination was recommended. For the period 2010 to 2014, ATAGI recommendations and funded program advocated for infant vaccination plus vaccination of household members and primary caregivers (cocooning). From 2015, the ATAGI recommendations and funding were updated to promote infant vaccination plus third trimester maternal immunization.^8^

#### Vaccine safety data

Yearly reported adverse event data were extracted from the “Database of Adverse Event Notifications (DAEN)- Medicines” for the DTPa-HBV-IPV/Hib vaccine over the 2009–2018 period.^17^ We considered for review the ten most frequently reported adverse events associated with a single medicine.

### Data analysis and reporting

Childhood vaccination coverage at 12 months of age was analyzed between 2009 and 2018 for all Australian children and in the Indigenous sub-group. Indigenous status on the Australian Immunisation Register is captured as “Indigenous” for individuals of Australian Aboriginal or Torres Strait Islander decent, “non-Indigenous”, or “unknown” as reported by the child’s carer to the Medicare health system or by the vaccination provider.^19^ Indigenous children are estimated to represent approximately 3% of the population from an annual birth cohort of approximately 300,000.^20^ We calculated the annual number of notified cases of poliomyelitis, tetanus, diphtheria, hepatitis B, Hib and pertussis in children under one year of age and in Australians of all ages between 2009 and 2018. Disease incidence per 100,000 total population and for children <12 months of age was calculated for the same period. In the case of hepatitis B, notifications in Australia are classified as either “newly acquired” or “unspecified”. Newly acquired infections are those for which there is serological or clinical evidence that infection occurred within the previous two years. Unspecified cases (without evidence of newly acquired infection) are more likely to represent those individuals who are newly diagnosed with chronic hepatitis B.^21^ For completeness, the total number of hepatitis B notifications (newly acquired plus unspecified) was used in our analysis.

Pertussis notifications by month of age in infants <12 months of age were used to calculate the incidence of disease across each month of life over the period 2006–2018. A surrogate “intention to treat” analysis was performed to calculate the incidence of pertussis according to the number of doses of vaccine each infant was eligible to have received at the time of their pertussis notification. The annual average (mean) and standard error of the mean (SEM) incidence of pertussis was calculated for infants eligible for zero (infants <2 months of age), one (infants two to four months of age), two (infants four to six months of age), and three doses (infants six to twelve months of age) between 2006 and 2018. The resulting data were then grouped according to the recommended pertussis vaccination practice as; (i) infant vaccination alone (2006–2009), (ii) infant vaccination plus post-partum parental vaccination or “cocooning” (2010–2014), and (iii) infant vaccination plus maternal vaccination during the second and third trimesters of pregnancy (2015–2018).

Spontaneously reported adverse events associated with DTPa-HBV-IPV/Hib as a single medicine were tallied from January 2009 to December 2018 and reported per 100,000 doses of vaccine (mean ± SEM), according to the total annual number of doses administered.

### Compliance with research ethics guidelines

This article is based on previously conducted studies as well as publicly available surveillance data and does not contain any new studies with human participants or animals performed by any of the authors.

## Results and discussion

### Childhood vaccination coverage in Australia

**Figure 1.**
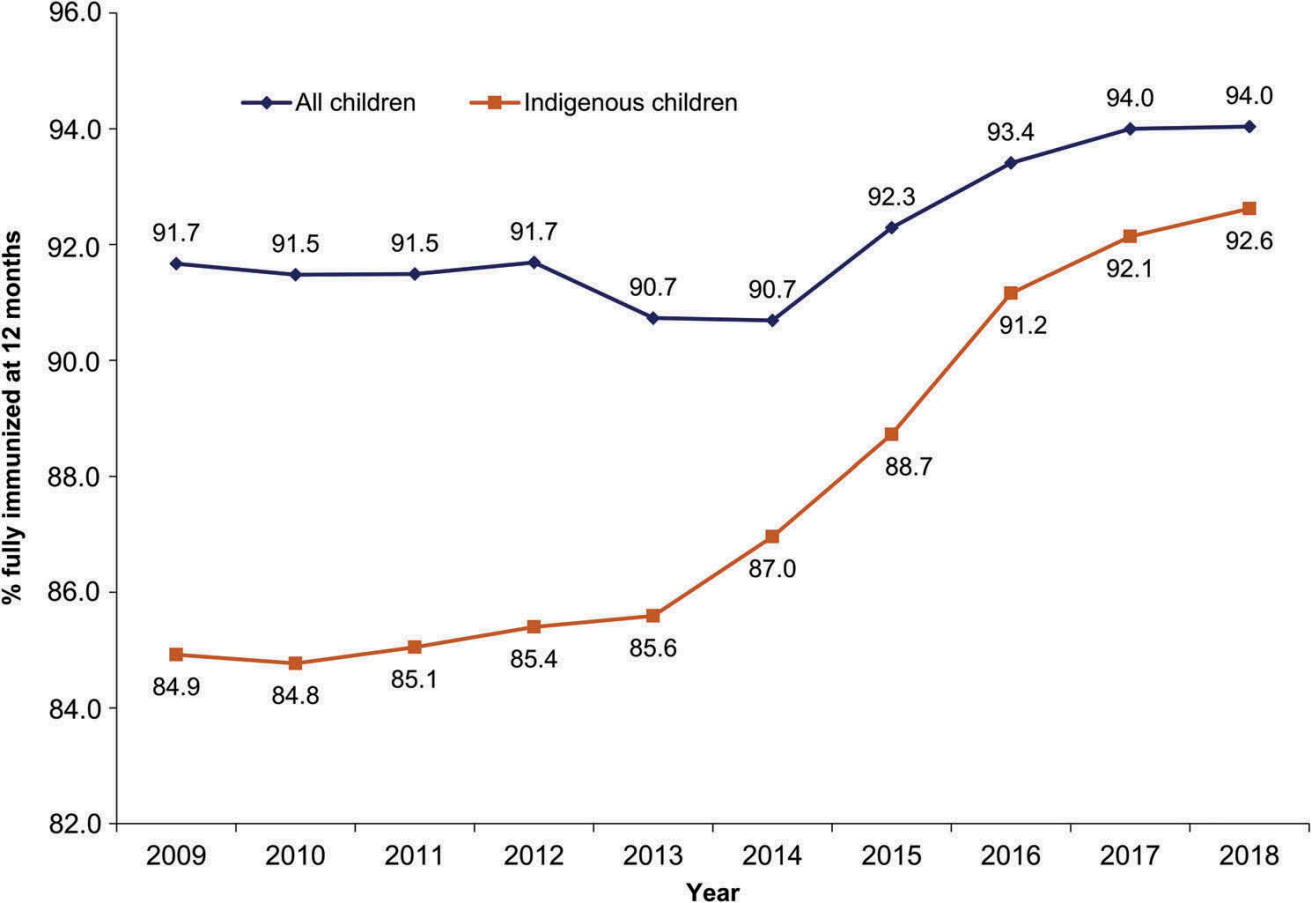
Coverage rates for all infants and Indigenous infants fully immunized* at 12 months of age over the 2009–2018 period in Australia

Since implementation of the DTPa-HBV-IPV/Hib vaccine in the NIP in 2009, full vaccination coverage rates for all infants aged 12 months, as measured through the AIR (previously the Australian Childhood Immunisation Register (ACIR)), have increased from 91.67% to 94.04% (Figure 1). Greater gains have been observed in the coverage rates of Indigenous children, from 84.2% in 2009 to 92.6% in 2018. Traditionally, Indigenous children have experienced a higher burden of vaccine preventable diseases than their non-Indigenous counterparts. Among the factors contributing to this disparity are lower socioeconomic status as well as increased likelihood of living in a remote or very remote geographical region.^20,22,23^ A national reform agreement to close the gap in Indigenous healthcare and disadvantage has contributed to improvements in vaccination coverage and outcomes for this population.^24,25^ There is evidence that when vaccine uptake is good, similar levels of disease control can be seen in the Indigenous population as in the general population.^23,26,27^

Footnote: *Received all doses for the diphtheria-tetanus-pertussis (DTP), poliomyelitis, *Haemophilus influenzae type b*, hepatitis B, and pneumococcal antigens recommended by 12 months of age. Data extracted from the Australian Immunisation Register.^12^

### Tetanus, diphtheria and poliomyelitis in Australian children

The incidence of tetanus in Australia has fallen since the introduction of diphtheria-tetanus-pertussis (DTP) vaccination in 1953, and remains very low, largely due to sustained high vaccination coverage.^28^ Only three cases of tetanus were reported in 2018 in Australia, none of which occurred in the infant population.^16^ Since the introduction of the combined DTPa-HBV-IPV/Hib vaccine, no cases of tetanus have been reported in infants less than one-year old.^16^

A combined toxin-antitoxin diphtheria vaccine was introduced in the 1920s for children, followed by the use of a diphtheria toxoid vaccine in a school-based program from 1932.^28^ The introduction of the DTP vaccine in 1953 saw continued decline in diphtheria incidence.^29,30^ Today, diphtheria is rare in Australia despite remaining endemic throughout much of the world (including parts of South East Asia).^31^ Between 2009 and 2018, no case of diphtheria was notified in infants less than one year of age.^16^ The few cases of diphtheria which are imported from overseas occur in individuals who have not been vaccinated.^32^ Use of the DTPa-HBV-IPV/Hib vaccine remains key to preventing the spread of diphtheria from imported cases.

The last locally acquired wild type poliomyelitis case in Australia occurred in 1972, with Australia officially declared polio-free by the World Health Organization in 2000.^33^ However, an imported case of wild poliovirus was reported in 2007,^30,34^ emphasizing the need for continued vaccination against poliomyelitis.

### Hepatitis B

Despite overall low prevalence of hepatitis B infection in Australia,^35^ Indigenous Australians have a markedly higher incidence, estimated to be as high as 17%, which is far above the globally recognized high-endemicity threshold.^36^ Hepatitis B screening was introduced for all pregnant women in Australia in 1985, allowing vaccination of infants born from mothers diagnosed with chronic hepatitis B.^21^ In 1990, vaccination was funded for all Northern Territory infants as a three-dose series, commencing at birth. A national vaccination strategy, which included a birth dose and three additional doses delivered at two, four, and six months, was adopted from 2000 onwards. The current NIP schedule still consists of a monovalent hepatitis B vaccine dose at birth, followed by the three DTPa-HBV-IPV/Hib doses.^10^

**Figure 2.**
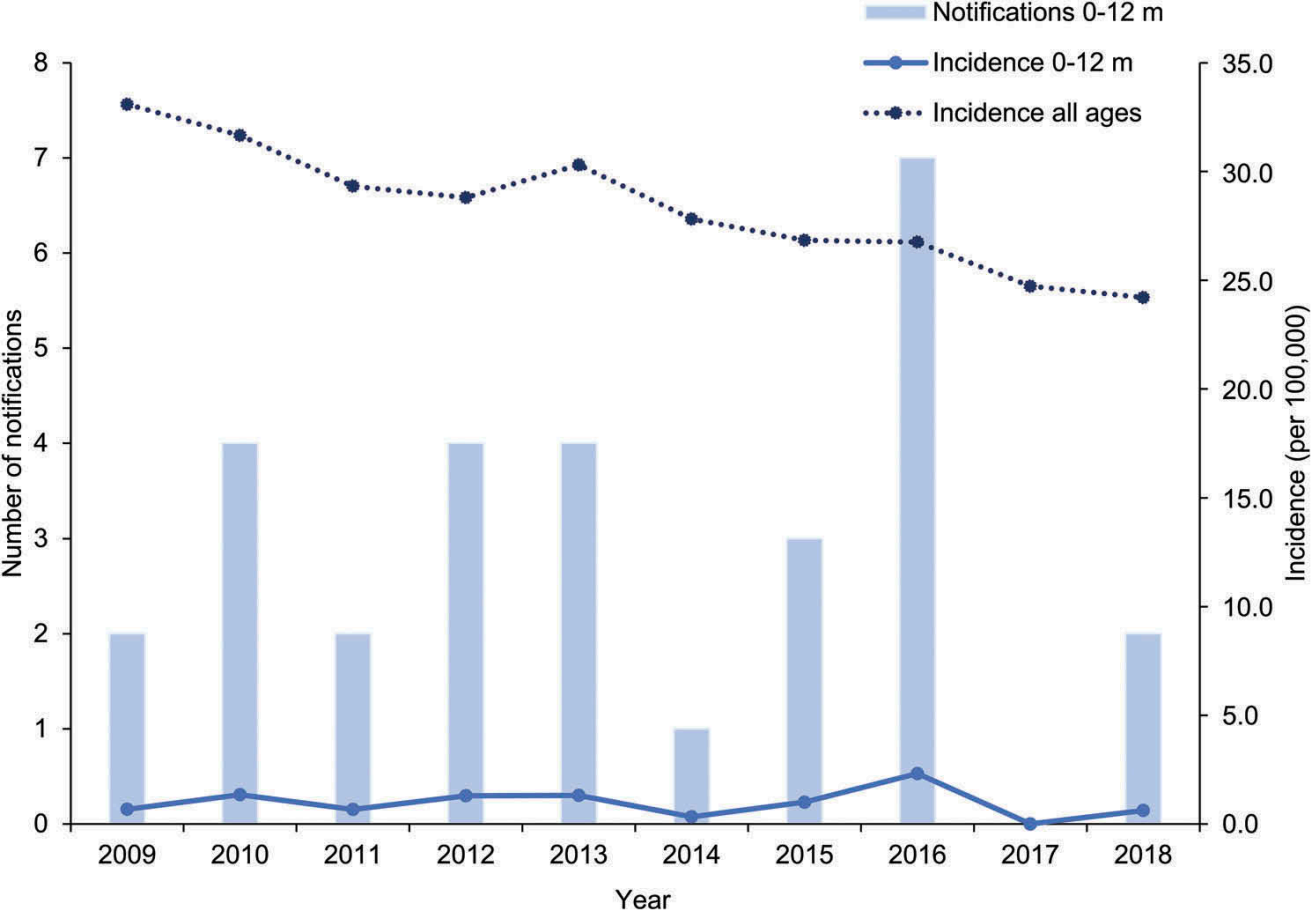
Number of notifications and incidence of hepatitis B in infants aged 0 to 12 months and incidence in all ages over the 2009–2018 period in Australia

The overall incidence of hepatitis B in Australia across all age groups decreased from 33.1 to 24.2 cases per 100,000 between 2009 and 2018 (Figure 2). Over the same period, the annual number of hepatitis B cases notified in infants less than one-year old remained below ten, leading to incidence rates ranging from 0.0 to 2.3 per 100,000.

Footnote: HepB, hepatitis B; m, months. Notifications are defined as laboratory-confirmed cases. Total number of notifications (newly acquired and not specified) were included. Data obtained from the National Notifiable Diseases Surveillance System.^16^

The frequency of mother to child transmission of hepatitis B in Australia has been evaluated by several studies. In 2009, Wiseman and colleagues assessed the perinatal transmission of hepatitis B from 313 pregnant women to their children.^37^ Among the 138 babies born to hepatitis B DNA-positive mothers, four cases of transmission were detected.^37^ Giles and colleagues conducted a retrospective assessment of the compliance with procedures for the prevention of mother-to-child transmission of hepatitis B among the 46,855 births having occurred in three maternity services across Victoria over the 2006–2011 period.^38^ They observed that 87% of the 398 hepatitis B-positive pregnant women were non-Australian-born.^38^ These statistics are supported by a recent report from the Kirby Institute, evidencing that while Asian migrants and Indigenous people together account for approximately 10% of the total Australian population, together they represent half of the Australian population living with chronic hepatitis B.^21^

Graham and colleagues reviewed the burden of chronic hepatitis B among Indigenous Australians prior to and following the introduction of the NIP schedule.^36^ Before 2000, the estimated prevalence of chronic infection among the adult Indigenous population was in excess of 16%, compared to just 0.4% of non-Indigenous adults.^36^ As of 2013, these rates were estimated to have reduced to 4% in Indigenous adults, compared to 0.9% of the non-Indigenous adult population.^39^ While vaccination has undoubtedly played a significant role in reducing this gap, there remains a need for opportunistic screening of at-risk Indigenous populations to assist with identification of those with chronic infection.^40^

Along with the delivery of a birth dose of hepatitis B vaccine within the first seven days of life, use of the combined DTPa-HBV-IPV/Hib vaccine remains critical for ensuring long-term immunity against the hepatitis B virus and prevention of chronic diseases in Australian infants. Recent data have confirmed persistence of immune response against hepatitis B 15 years following infant vaccination,^41^ demonstrating the ongoing value of vaccination of this population.

### Haemophilus influenzae *type b*

Pre-vaccination estimates for incidence of *Haemophilus influenzae* type B (Hib) meningitis in children aged less than five years in Australia were 26 and 150 cases per 100,000, in non-Indigenous and Indigenous children, respectively.^42–44^ Case-fatality rates and severe sequelae after Hib meningitis episodes were also higher in Indigenous than in non-Indigenous people, further emphasizing the burden of Hib in this population.^44^

Hib conjugate vaccines were first added onto the Australian vaccination schedule in 1993.^45^ Conjugation of the Hib polyribosylribitol phosphate (PRP) capsule to a carrier protein component ensures generation of a T cell-dependent immune response in children less than two years of age.^46–48^ Between 1993 and 2000 there were different Hib vaccination schedules in place for Indigenous and non-Indigenous children (Table 1).^49^ Non-Indigenous infants received a tetanus toxoid (TT)-conjugated vaccine, while Indigenous children and all infants in the Northern Territory received a *Neisseria meningitidis* outer-membrane protein (OMP)-conjugated Hib vaccine.^8,50^ Compared to TT and diphtheria toxoid (DT)- and CRM-conjugation, OMP-conjugation of the PRP capsule had been demonstrated to elicit a rapid immune response following the first dose of vaccine in Indigenous children in Alaska.^51^

In March 2000, the vaccination schedule was modified so that all Australian children would receive the OMP-conjugated vaccine, achieving the important end-point of standardized care across the entire population.^49^ Further refinement of the Hib schedule occurred between 2005 and 2009 with progressive phasing from OMP to TT-conjugated vaccine and the inclusion of DTPa-HBV-IPV/Hib vaccine on the national schedule.^52^ TT-conjugated vaccines remain favored due to their superior immune persistence compared to OMP-conjugated variants.^48,53,54^ The inclusion of a Hib booster vaccination during the second year of life has also remained critical to ensuring optimal long-term protection of infants against disease.^55^

The Hib component of the DTPa-HBV-IPV/Hib vaccine is presented as a lyophilized powder that requires reconstitution prior to administration.^7^ Separation of the Hib component achieves two important goals; (i) maintenance of vaccine antigen stability throughout the shelf life of the vaccine by removing the risk of depolymerization,^56,57^ therefore (ii) ensuring lot-to-lot consistency of the Hib component.^7^

**Figure 3.**
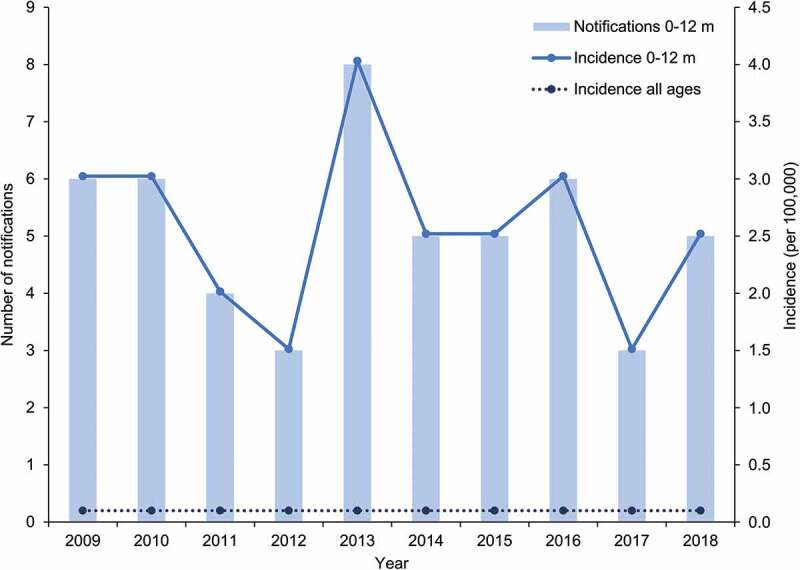
Number of notifications and incidence of *Haemophilus influenzae* type B (Hib) in infants aged 0 to 12 months and incidence in all ages over the 2009–2018 period in Australia

Between 2009 and 2018, the average annual number of Hib notifications reported in all age groups was 18, leading to incidence rates lower than 0.1 per 100,000.^16^ Over the same period an average of five Hib notifications were reported in infants aged less than one year annually, leading to incidence rates varying between 1.5 and 4.0 per 100,000 (Figure 3). Of those cases occurring within the first 12 months of life, an average of just three cases per year were seen during the first six months of life. None of the notifications observed over this period represented outbreaks of invasive Hib disease.

Footnote: m: months. Notifications are defined as laboratory-confirmed cases. Data obtained from the National Notifiable Diseases Surveillance System.^16^

An analysis of the 54 cases of Hib notified between 2014 and 2016 reported nine (16.7%) occurred in Indigenous people although they were estimated to represent only 3.3% of the total population.^17^^,30^ In a local review on the impact of Hib vaccination, Menzies *et al*. suggested that the increased burden in Indigenous children may be the combined result of earlier exposure to the bacteria and more persistent nasopharyngeal colonization, as well as various factors contributing to suboptimal immunologic response to vaccination, including delayed receipt of the primary immunization series.^58^ In line with this, the incidence of invasive Hib disease over the 1995–2005 period in Indigenous children under five years of age was consistently observed to be three- to 17-fold higher than in non-Indigenous children.^45^ Further, numbers of Hib cases reported in Australian children aged less than five years old between 2007 and 2010 also showed a disparity with annual incidence rates of 5.6 and 0.4 per 100,000 in Indigenous and non-Indigenous children, respectively.^59^ However, in the context of the small proportion of the total birth cohort, this represents an average of less than two notifications annually, suggesting that Hib disease remains well controlled within the Indigenous population, even within smaller remote and highly remote communities.^52^ Unlike elsewhere in the world, there has been no documented resurgence or return of invasive Hib disease following the switch from OMP to TT-conjugated vaccines in the Australian Indigenous population.^52^ Rather, continued disparities in disease burden between Indigenous and non-Indigenous populations highlight the importance of achieving high uptake and timely delivery of vaccines as well as the reduction of other modifiable risks for vaccine-preventable disease in at-risk populations.

### Pertussis

In Australia, pertussis vaccination was first introduced in the 1940s and the combined diphtheria-tetanus-pertussis vaccine was introduced in 1953.^28,60^ Following recommendation from the National Health and Medical Research Council, whole cell vaccines were replaced by acellular vaccines in 1999.^61^ The current childhood NIP schedule includes vaccination against pertussis at two, four, and six months of age.^10^ Local guidelines have included a recommendation to commence this schedule at six weeks of age rather than at eight weeks as a means of providing earlier protection against severe disease.^8^ The primary DTPa-HBV-IPV/Hib series is followed by booster doses of DTPa vaccine at 18 months and four years.^10^ A further booster with a reduced antigen content vaccine (dTpa) is delivered at 12–13 years of age.^10^ Maternal dTpa vaccination is also provided under the Australian NIP during every pregnancy from 20 weeks.^8,10^ These vaccination strategies are geared toward controlling the severity of disease in the youngest age groups.

Pertussis notifications follow a cyclical pattern in Australia, with seasonal peaks occurring every three to four years.^62,63^ In recent years, larger and more sustained peaks have been observed and these are thought to be due to several factors. Improved availability and testing methods, such as polymerase chain reaction, to confirm suspected cases, as well as increased access to primary care services, have contributed to an increase of pertussis notifications.^64^ Heightened awareness of pertussis by the medical and general communities has also led to more frequent testing.^64^ These factors together likely represent an improvement in the ability to measure the true burden of pertussis in the community,^65,66^ however, it is important to recognize that a large proportion of pertussis infections, particularly those affecting adults still go undiagnosed.^66^

**Figure 4.**
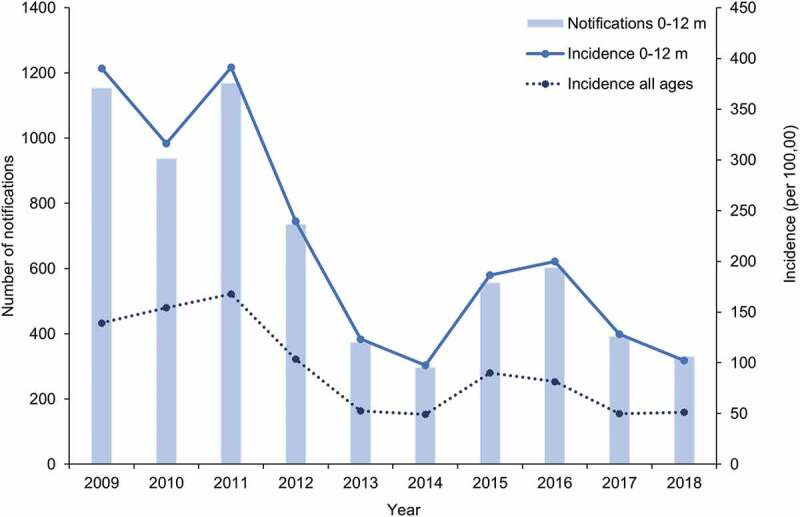
Number of notifications and incidence of pertussis in infants aged 0 to 12 months and incidence in all ages over the 2009–2018 period in Australia

Vaccination programs, together with advances in healthcare, have had a considerable impact on pertussis-related mortality.^30,60^ Whilst pertussis death is now rare, severity is still of concern in infants under one year of age.^67–69^ Review of the data collected between 1994 and 2004 in Australia found that infection is most likely to occur in infants who are too young to be fully immunized or had not received timely vaccination.^70^ The incidence of pertussis in infants less than one year of age remains approximately double the rate seen in the overall population (Figure 4). Incidence data collected over the 2009–2018 period for children under one year of age show an overall decreasing trend from 390 to 102 cases per 100,000 individuals.

Footnote: m: months. Notifications include confirmed and probable cases. Data obtained from the National Notifiable Diseases Surveillance System.^16^

**Figure 5.**
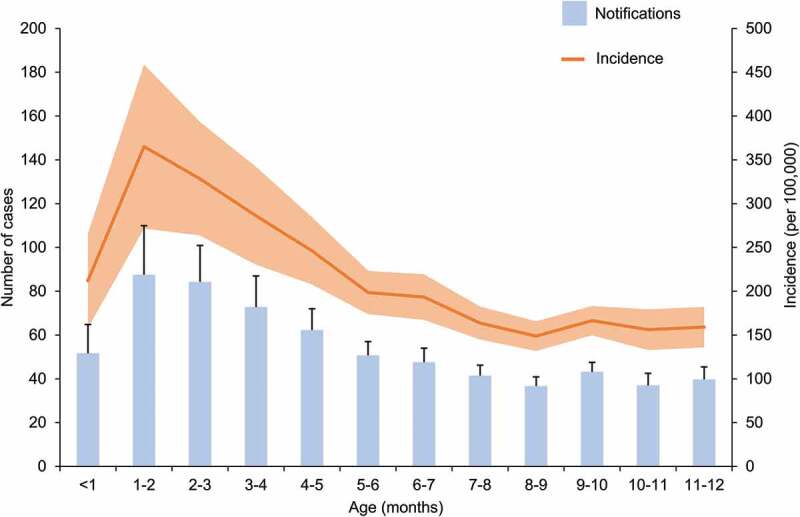
Average notifications and incidence of pertussis by month of age during first year of life, over the 2009–2018 period in Australia

The Pediatric Active Enhanced Disease Surveillance (PAEDS) network has reported pertussis notifications in children under 15 years of age since 2012. The aim of this surveillance is to determine the factors contributing to hospitalization and severe outcomes due to pertussis (and other vaccine preventable diseases) at specific sentinel sites across Australia.^71^ In 2015 and 2016, 42% and 23% of all children hospitalized with pertussis were aged less than three months, respectively.^72,73^ Over the 2012–2015 period, 37% of the children under one year of age who were hospitalized with laboratory confirmed pertussis were too young to be vaccinated.^72^ In line with these findings, NNDSS data collected between 2009 and 2018 demonstrate a peak in the average incidence of pertussis at approximately two months of age (Figure 5). As the first dose of pertussis vaccine cannot be administered earlier than six weeks of age,^7^ additional strategies have been sought to assist with control of pertussis during the first months of life.

Footnote: Error bars (notifications) and light orange (incidence) represent the standard error to the mean. m: months. Notifications include confirmed and probable cases. Data obtained from the National Notifiable Diseases Surveillance System.^16^

### Strategies for reducing burden of pertussis disease in early infancy

Recently, three strategies aimed at reducing the burden of pertussis disease in infants during their first months of life have been introduced in Australia.^67–69^

#### Optimization of the childhood vaccination schedule

As early as 2011, Foxwell and colleagues estimated an 8% reduction of pertussis notifications if the first dose of vaccine was administered at six weeks instead of two months of age.^74^ In line with this, early administration of the first DTPa-HBV-IPV/Hib dose, at six, as opposed to eight weeks of age has been encouraged in Australia since 2011.^75^

In 2016, an increase in the number of notifications of pertussis in children aged two to four years old led to the re-introduction of the 18-month booster dose.^76^ This booster dose, delivered as DTPa, was initially included in the NIP schedule but removed in 2003. Subsequently, the ATAGI recommended the re-introduction of this pertussis booster dose at 18 months necessary to improve pertussis control and limit the transmission to infants from older siblings.^76^

#### Cocooning

As the source of infant pertussis infection can commonly be traced to close relatives,^77^ vaccination of adult household members has been suggested as an effective way to prevent infant pertussis notifications. In 2013, a review of nine studies by Wiley and colleagues reported when identified, the source of pertussis infection in hospitalized infants aged less than six months was the mother in 39% of cases.^78^ Fathers and grandparents accounted for 16% and 5% of the identified sources of infection, respectively.^78^

In Australia, post-partum parental vaccination was recommended and funded in various forms from 2010 to 2015.^8,10^ A case control study reported by Quinn and colleagues demonstrated that vaccination of both parents reduced the risk of pertussis infection for children less than four months of age by 51%.^79^ Notably, in order to be effective, immunization had to occur more than four weeks prior to infant disease onset. A similar study conducted by Rowe et al. reported a 77% reduction in pertussis in children less than one-year-old when both parents were immunized more than 28 days before infant disease onset.^80^ While there are local reports of high uptake of parental vaccination in this setting,^81,82^ the cocooning strategy is limited by the challenge of ensuring vaccination of all individuals that may potentially come into contact with a young infant.^83^ For this reason, maternal vaccination is favored as the primary strategy for the protection of new-born infants against pertussis due to its efficacy and logistical advantages.^70,84,85^

#### Maternal vaccination

During *in utero* development, antibodies are transferred across the placenta from the mother to the fetus.^86,87^ These antibodies have been demonstrated to provide passive immunity against influenza and pertussis to new-born infants during their first months of life.^88,89^ Maternal influenza vaccination was first introduced and funded under the NIP in Australia in 2010.^90^ Maternal dTpa vaccination during the third trimester of pregnancy was first recommended by the ATAGI in March 2015 and funded through state-based vaccination programs.^91,92^ The program has matured through inclusion on the NIP in 2018 and recent expansion of the recommendation for vaccination to occur at any time during the second and third trimesters.^8,93^

**Figure 6.**
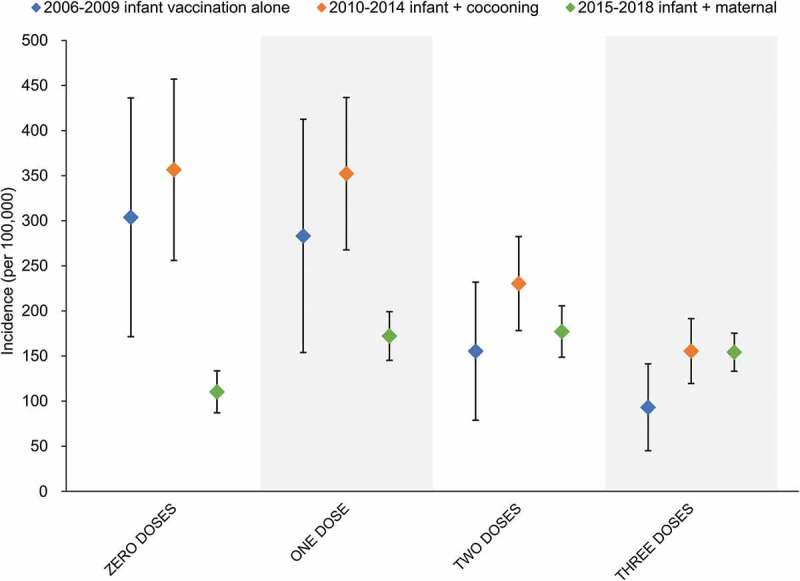
Average incidence of pertussis by number of vaccine doses received over the period of use of infant vaccination only and in combination with cocooning or maternal vaccination

Uptake of maternal pertussis vaccine in Australia has been reported to range between 64%,^94^ and 81%^95^ when included as standard antenatal care. The impact of maternal vaccination in reducing early pertussis notifications has been demonstrated both in Australia^96^ and overseas.^97,98^ This is further evidenced when NNDSS data are stratified according to the eligible number of doses of pertussis containing vaccine by age (Figure 6). The maternal vaccination era (2015–2018) has demonstrated significant reductions in the incidence of pertussis in infants who are too young to be eligible for receipt of the first dose of DTPa-HBV-IPV/Hib vaccine, compared to the cocooning (2010–2014) and infant alone (2008–2009) vaccination strategies. These differences are negated following receipt of the first DTPa-HBV-IPV/Hib dose, highlighting the continued importance of achieving high vaccine coverage and timely delivery of doses to maintain control of pertussis during the first year of life.

Footnote: Error bars represent the standard error to the mean. “Zero doses” refers to infants <2 months of age. “One dose” refers to infants 2–4 months of age. “Two doses” refers to infants 4–6 months of age. “Three doses” refers to infants 6–12 months of age. Cocooning refers to the post-partum parental vaccination. Maternal vaccination refers to maternal dTpa vaccination during the second and third trimesters of pregnancy. Data obtained from the National Notifiable Diseases Surveillance System.^16^

### Timeliness of childhood vaccination

As already discussed, childhood vaccination coverage rates in Australia are high and continuously improving (Figure 1). However, coverage rates are not the sole factor impacting the performance of vaccination programs. Timeliness of vaccination, i.e. age-appropriate administration of the vaccine dose, is of key importance for diseases that can severely impact infants, such as Hib and pertussis. The AIR provides an ideal tool to assess the timeliness of infant vaccination.^14^ Data collected between 1996 and 2012 reported that the proportion of children with delayed vaccination was higher in Indigenous children compared to non-Indigenous children (23.0% vs 11.9% for the first DTP containing dose, respectively).^99^ Significant inroads have been made in closing this gap with the latest National Center for Immunisation Research and Surveillance data demonstrating timely administration of the first DTPa dose now achieving 97.6% and 93.4% in non-Indigenous and Indigenous children, respectively.^100^ Timeliness of the second and third doses of DTPa falls to 91.1% and 81.5% in non-Indigenous children and to 80.0% and 65.8% in Indigenous children, respectively.^100^ This gap might partly explain the increased incidence of vaccine preventable diseases in Indigenous infants.^101^

Even though vaccination coverage rates in Australia are high, there is room for improvement of vaccination timeliness, particularly for encounters beyond the primary infant series (beyond the scope of the current review).^99,102^ Regarding the primary series, children born overseas or in remote areas of Australia are more likely to be delayed in their receipt of vaccinations.^103^ Delayed or missing vaccination is mainly due to parents’ lack of access to health services and other socioeconomic factors, such as larger family size and unemployment.^102,104,105^ Parental conscientious objection to vaccination is relatively rare in Australia, estimated at less than 2%,^106^ and therefore unlikely to play a significant role in determining vaccine uptake.^107^ Therefore, catch-up programs are the most efficient strategies for addressing the vaccination coverage and timeliness gaps in Australian children,^108^ and should ideally be initiated as early as possible to minimize the number of missed vaccination encounters. The upper age limit for administration of combination vaccines as well as the co-administration of multiple vaccines should be considered when planning a catch-up schedule.

### Comparison with other countries

Direct comparisons of Australian data with other developed countries are difficult due to variations in vaccines selection, schedule, program implementation, and surveillance. However, some broad comparisons and general trends are discussed below. Childhood vaccination coverage rates are generally high in other developed countries.^109^ However, a decreasing trend in coverage for vaccines against diseases including poliomyelitis, diphtheria, tetanus, and pertussis has been reported during the last decade in Europe.^110^ The percentage of European children aged two years old who received three doses of the combined DTP vaccine increased from 96.8% in 2009 to 97.5% in 2012, and then decreased to 95.1% in 2017.^110^ In the US, coverage of the third DTPa dose remained stable, at 94.1% in 2013, and 94.0% in 2017.^111^

Similarly, to Australia, the US and Europe have been declared polio free.^112,113^ Though some countries are of special concern to the European Regional Certification Commission (RCC) for Poliomyelitis Eradication due to their suboptimal vaccination coverage rates and weaknesses in disease surveillance.^112^ Tetanus and diphtheria cases are likewise rare in Europe and the US.^114,115^ Reported tetanus cases predominantly affect older, unvaccinated adults.^116,117^ Overall Hepatitis B incidence is low (<0.5/100,000) in children living in the US and in Europe.^118,119^ A review from 10 European countries that almost exclusively used the DTPa-HBV-IPV/Hib vaccine between 2006 and 2013 showed sustained control of Hib infection, suggesting vaccine effectiveness in these populations.^55^ Pertussis remains a concern in Europe as well as in the US, with an increasing trend in notifications and the highest burden in those aged less than one year.^120,121^

Finally, a review assessing immunogenicity and safety of the DTPa-HBV-IPV/Hib vaccine after eight years of licensure in Europe has confirmed vaccine impact and safety using post-marketing surveillance data.^5^ This adds to the body of evidence collected through multiple clinical trials, having studied or used as comparator the DTPa-HBV-IPV/Hib vaccine, that showed its acceptable safety profile.^122–124^

### Safety surveillance

**Figure 7.**
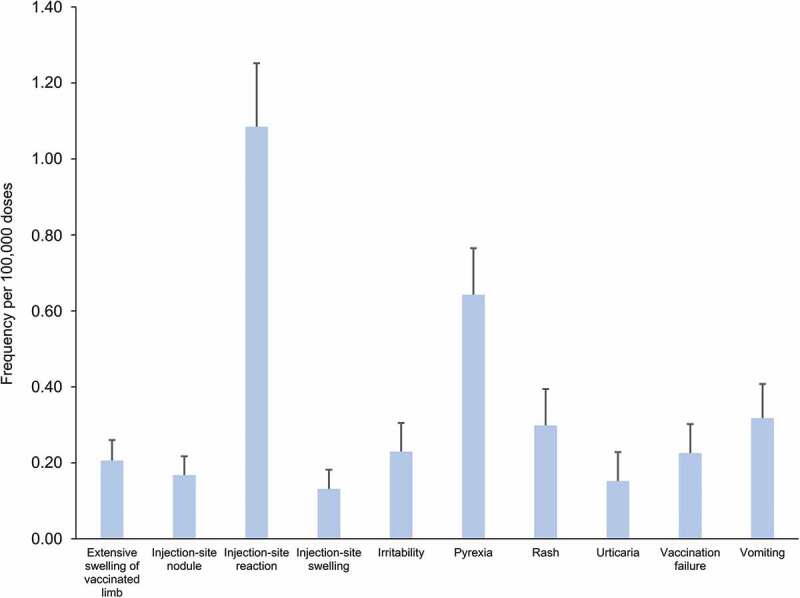
Frequency of adverse events reported for the use of DTPa-HBV-IPV/Hib vaccine as single medicine over the 2009–2018 period in Australia

Between 1 January 2009 and 31 December 2018, more than 9.6 million doses of DTPa-HBV-IPV/Hib vaccine were distributed on the Australian market (internal communication). Over the same period, a total of 9,066 adverse events were reported to the DAEN, among which 627 were suspected to have been caused solely by the administration of DTPa-HBV-IPV/Hib vaccine.^17^ The adverse event most frequently reported after administration of the vaccine was injection-site reaction (n = 105), followed by fever (n = 62), and vomiting (n = 30). The annual frequency of the ten most commonly reported adverse events per 100,000 doses (Figure 7) is consistent with the safety profile reported for the DTPa-HBV-IPV/Hib vaccine in clinical trials and the product information,^7,125^ as well as in other safety surveillance data.^5,126^

Footnote: Error bars represent the upper limit of the standard error to the mean. DTPa-HBV-IPV/Hib: combined vaccine against diphtheria, tetanus, pertussis, hepatitis B, poliomyelitis, and *Haemophilus influenzae* b. Data extracted from the Database of Adverse Event Notifications.^17^

### Limitations and caveats of the current review

National databases have been used for the collection of data relating to vaccine coverage rate, disease notifications and adverse events following vaccine administration. These data are assumed to accurately reflect childhood vaccination with the DTPa-HBV-IPV/Hib vaccine. Nevertheless, there are some limitations worth highlighting.

Firstly, vaccination coverage rates are reported throughout this review according to receipt of all the vaccines recommended by 12 months of age for the one-year-old cohort (children aged 12 to less than 15 months). This means that an individual has received all doses for the DTP, poliomyelitis, Hib, hepatitis B, and pneumococcal antigens according to the Australian Handbook recommendations at that timepoint. While tables detailing the current year (12-month period) provide the break down across all vaccine types/antigens, historical tables provide only an overall coverage rate which includes children having been vaccinated against all diseases before they reached 15 months of age. It is therefore possible that historical coverage rates underestimate the number of infants who have completed a full schedule of DTPa-HBV-IPV/Hib vaccine through exclusion of those who did not receive one or more of the other vaccines during their first year of life. Moreover, there is currently limited publicly reportable information available from the AIR regarding the timeliness of vaccine administration, despite this being a critical factor in childhood vaccination, particularly for optimal protection against pertussis.

Secondly, surveillance data reporting vaccine-preventable disease notifications rely on computerized, completely de-identified records of notifications being provided by State/Territory health authorities under the provisions of the public health legislation in their jurisdiction.^16^ This de-identification prohibits the analysis of disease incidence and its correlation with receipt of appropriate vaccine/s for any single individual. Likewise, publicly available historical tables do not include information on the Indigenous status of notifications.

Finally, the current safety analysis assumes that all serious adverse events occurring after administration of the vaccine are reported to the TGA. Where the appropriate reporting processes are not followed and that adverse events may be omitted from TGA reports extracted from the DAEN. The potential underestimation of adverse events is expected to be low as reporting failure is anticipated to occur infrequently. Further, the frequency of adverse events presented in this review is calculated for those relating to a single suspected medicine, i.e. DTPa-HBV-IPV/Hib vaccine only. Those events observed in the setting of co-administered medicines have been omitted. As the Australian Immunisation Handbook encourages co-administration of multiple funded and private-market vaccines with the first two doses of DTPa-HBV-IPV/Hib vaccine, some adverse events might have been notified for all injected vaccines. This may, in turn, lead to an underestimation of the frequency when looking at the number of reports related to a single suspected medicine. However, frequencies based on all reports would have included adverse events more typically reported for other vaccines or resulting from the co-administration which was not the focus of the current analysis.

## Conclusion

The DTPa-HBV-IPV/Hib vaccine has demonstrated beneficial impact in Australia since its introduction in 2005, maintaining protection of infants against diphtheria, tetanus and poliomyelitis, and improving protection against hepatitis B, pertussis and Hib, particularly in Indigenous infants. Timely administration of DTPa-HBV-IPV/Hib vaccine remains key to maintaining control of pertussis. Due to their vulnerability, Australian infants aged less than 12 months of age, in particular those too young to be vaccinated, remain the most vulnerable to pertussis. Additional strategies, such as maternal vaccination and administration of the first vaccine dose at six weeks have had a beneficial impact on the incidence of pertussis in newborns, reducing the number of notifications in the first six months of life.

Safety monitoring carried out since the introduction of DTPa-HBV-IPV/Hib over a ten-year period provides confidence about the reliability of its safety profile.

**Figure 8.**
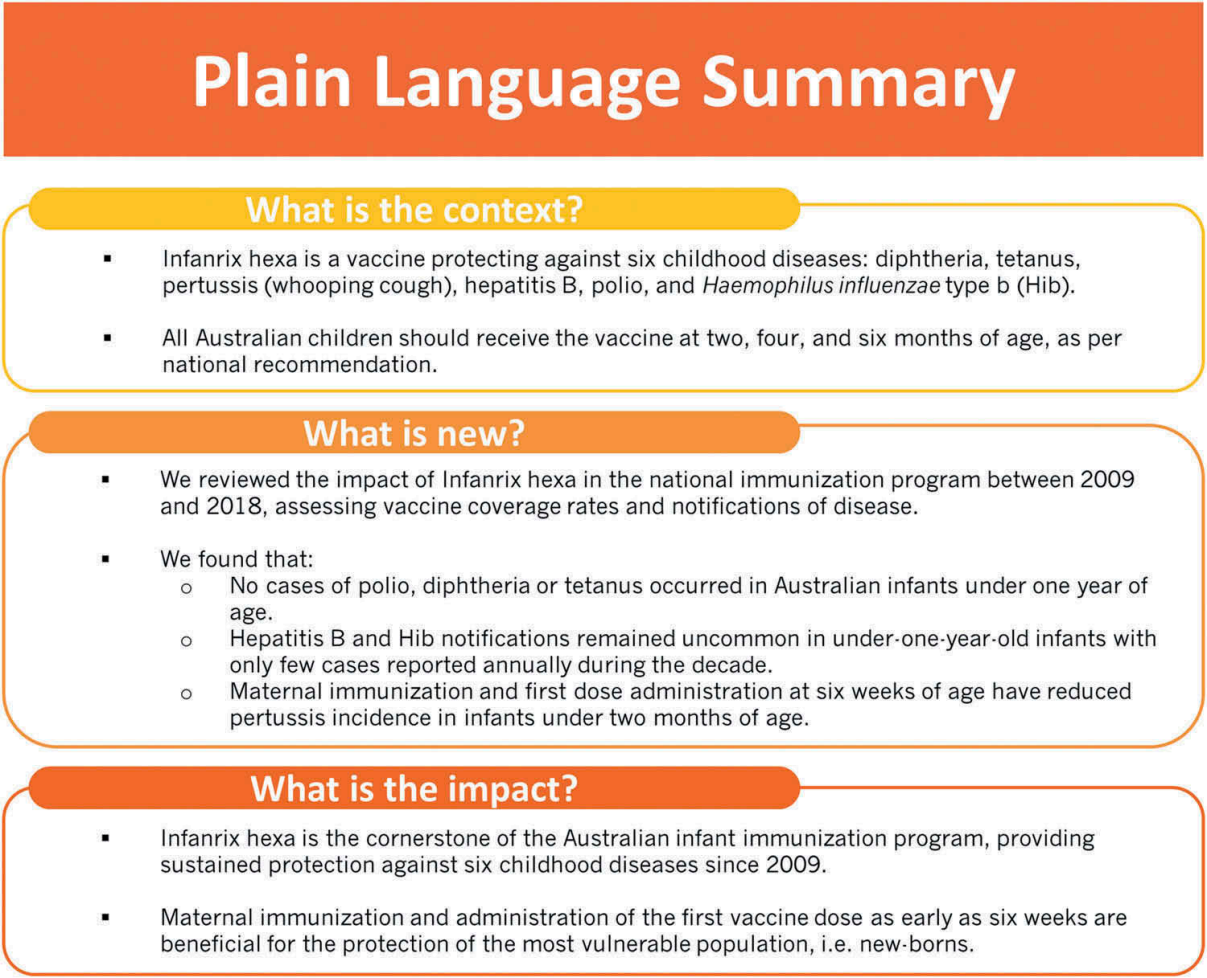
Plain language summary

The use of DTPa-HBV-IPV/Hib together with its high vaccine coverage is the foundation of the Australian NIP, providing a solid base for protection of children against childhood infectious diseases (Figure 8). Use of a combined vaccine protecting against six childhood diseases has an even greater impact where remoteness and healthcare access stands in the way of vaccination.

## Data Availability

The datasets generated during and/or analyzed during the current study have been made available to the authors following specific request to the Office of Health Protection. Summarized data are publicly available from the Communicable Diseases Network Australia on reasonable request.

## Medical writing, editorial, and other assistance

The authors thank Business & Decision Life Sciences platform for editorial assistance and manuscript coordination, on behalf of GSK: Jonathan Ghesquiere provided writing support and Janne Tys coordinated manuscript development and editorial support.
